# *CELSR3* variants are associated with febrile seizures and epilepsy with antecedent febrile seizures

**DOI:** 10.1111/cns.13781

**Published:** 2021-12-23

**Authors:** Jia Li, Si‐Mei Lin, Jing‐Da Qiao, Xiao‐Rong Liu, Jie Wang, Mi Jiang, Jing Zhang, Min Zhong, Xu‐Qin Chen, Jing Zhu, Na He, Tao Su, Yi‐Wu Shi, Yong‐Hong Yi, Wei‐Ping Liao, for the China Epilepsy Gene 1.0 Project

**Affiliations:** ^1^ Institute of Neuroscience and Department of Neurology of the Second Affiliated Hospital of Guangzhou Medical University Key Laboratory of Neurogenetics and Channelopathies of Guangdong Province and the Ministry of Education of China Guangzhou China; ^2^ Department of Pediatrics Xiangya Changde Hospital Changde China; ^3^ Department of Neurology Children’s Hospital of Chongqing Medical University Chongqing China; ^4^ Department of Neurology Children’s Hospital of Soochow University Suzhou China; ^5^ Department of Pediatrics The First Hospital of Anhui Medical University Hefei China

**Keywords:** *CELSR3*, epilepsy, febrile seizures, pathogenic gene, trio‐based whole‐exome sequencing

## Abstract

**Aims:**

To identify novel pathogenic gene of febrile seizures (FS)/epilepsy with antecedent FS (EFS+).

**Methods:**

The trio‐based whole‐exome sequencing was performed in a cohort of 462 cases with FS/EFS+. Silico programs, sequence alignment, and protein modeling were used to predict the damaging of variants. Statistical testing was performed to analyze gene‐based burden of variants.

**Results:**

Five heterozygous missense variants in *CELSR3* were detected in five cases (families) with eight individuals (five females, three males) affected. Two variants were de novo, and three were identified in families with more than one individual affected. All the variants were predicted to be damaging in silico tools. Protein modeling showed that the variants resulted in disappearance of multiple hydrogen bonds and one disulfide bond, which potentially caused functional impairments of protein. The frequency of *CELSR3* variants identified in this study was significantly higher than that in controls. All affected individuals were diagnosed with FS/EFS+, including six patients with FS and two patients with EFS+. All cases presented favorable outcomes without neurodevelopmental disorders.

**Conclusions:**

*CELSR3* variants are potentially associated with FS/EFS+.

## Graphical abstract

Five heterozygous missense variants in *CELSR3* are detected in five unrelated cases from a cohort of patients with febrile seizures (FS)/epilepsy with antecedent FS (EFS+) by performing trios‐based whole‐exome sequencing. This study suggests that *CELSR3* is potentially a candidate‐causative gene of FS/EFS+

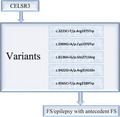

## 1 INTRODUCTION

Febrile seizures (FS) are the most common convulsive event in humans. Generally, about 4%–5% of population suffer at least one FS during lifetime, whereas the incidence of FS in the Asian population is up to 8%–10%.^1^, ^2^ FS alters susceptibility of nervous system under exposure to fever.^3^ The biological basis of FS remains unknown. Increasing evidence supports that genetic factor is a predominantly pathogenic element of FS.^2^, ^4^, ^5^ FS is a kind of epileptic seizure by nature, and children with FS are five times more likely to develop subsequent unprovoked seizures (epilepsy with antecedent FS, EFS+) compared with children without FS.^2^ So far, the identified genes that causes FS/EFS+include *ADGRV1*, *CPA6*, *DYRK1A*, *FEB2*, *FEB5*, *FEB6*, *FEB7*, *FEB9*, *FEB10*, *FGF13*, *GABRB3*, *GABRD*, *GABRG2*, *GEFSP4*, *GEFSP6*, *GEFSP8*, *HCN1*, *NPRL3*, *SCN1A*, *SCN1B*, *SCN2A*, *SCN9A*, and *STX1B* (OMIM, https://www.omim.org/). However, the genetic causes of the majority patients with FS/EFS+ are unknown.

*CELSR3* (OMIM* 604264) encodes cadherin EGF LAG seven‐pass G‐type receptor 3 (CELSR3), which is a special subgroup of adhesion G protein‐coupled receptors that is expressed mainly in the brain across whole lifespan.^6^ Although function of *CELSR3* is uncertain, previous studies have shown that *CELSR3* plays a crucial role in dendrite development, axon guidance, and brain wiring.^6^, ^7^, ^8^, ^9^ Mice of homozygous *CELSR3* null exhibit neonatal lethality and abnormal nervous system development, such as abnormal morphology of embryonic or fetal subventricular zone, globus pallidus malformation, anomalous innervation, and neuronal migration disorder.^10^, ^11^ These findings suggest an essential role of *CELSR3* in neurodevelopment. *CELSR3* variants have been occasionally detected in various neurological diseases,^12^, ^13^, ^14^, ^15^, ^16^, ^17^, ^18^, ^19^, ^20^, ^21^ including autism spectrum disorder, developmental delay, epileptic encephalopathy, intellectual disability, neural tube defects, and Tourette syndrome. However, the association between *CELSR3* variants and human diseases is not determined.

In this study, we performed trios‐based whole‐exome sequencing (WES) in a cohort of patients with FS/EFS+ to screen novel genetic variants. Five heterozygous missense variants in *CELSR3* were detected in five unrelated cases. This study suggests that *CELSR3* is potentially a candidate causative gene of FS/EFS+.

## 2 MATERIALS AND METHODS

### 2.1 Subjects

We recruited the patients with FS/EFS+ from five hospitals in five regions in China, including The Second Affiliated Hospital of Guangzhou Medical University (Guangzhou), Children's Hospital of Chongqing Medical University (Chongqing), Children's Hospital of Soochow University (Suzhou), The First Hospital of Anhui Medical University (Hefei), and Xiangya Changde Hospital (Changde). Epileptic seizures and epilepsy syndromes were diagnosed according to the criteria of the Commission on Classification and Terminology of the International League Against Epilepsy (1981, 1989, 2001, 2010, and 2017). Focal epilepsy was used to denote patients with focal seizures, such as focal motor seizures, focal non‐motor seizures, and focal to bilateral tonic‐clonic seizures, supported by unifocal/multifocal epileptiform discharges on electroencephalogram (EEG). Generalized epilepsy was diagnosed based on a range of seizure types including absence, myoclonic, atonic, tonic, and tonic‐clonic seizures. FS was diagnosed with the criteria^22^, ^23^: (1) a seizure occurring in childhood after age of 1 month to 5 years accompanied by a fever, (2) the febrile illness not caused by central nervous system infection, (3) not meeting criteria for other acute symptomatic seizures. Patients with FS or epilepsy with antecedent FS were included. The exclusion criteria were patients with brain structural abnormalities and epilepsies with acquired causes. Clinical information of cases was collected, including age of onset, characteristics of seizures, history of afebrile seizures, state of development, family history, and response to anti‐seizure medications (ASMs). Brain magnetic resonance imaging (MRI) scans, including T1‐weighted (morphology), T2‐weighted, and FLAIR/T1 (intensity) features, were performed. The images of standard sagittal, coronal, and axial views were obtained for detecting the structural abnormalities. Long‐term (24 h) video‐EEG monitoring records were performed with electrodes being arranged according to the international standard of 10–20 reduced montage system. The procedures of open‐close eyes test, hyperventilation, intermittent photic stimulation, and sleeping recording were obtained. Eventually, a total of 462 cases were enrolled, including 285 patients with FS, 92 patients with generalized epilepsy with antecedent FS, and 85 patients with focal epilepsy with antecedent FS.

This study complied with the principles of the International Committee of Medical Journal Editors with regard to patient consent for research or participation. Written informed consent was obtained from the individuals or legal guardians. Ethical approval had been obtained from Ethics Committee of the Second Affiliated Hospital of Guangzhou Medical University.

### 2.2 WES and bioinformatic analysis

The blood samples were collected from the probands, their parents, and other available family members to determine the origin of the identified genetic variants. The genomic DNAs from the blood samples were extracted by using the Qiagen Flexi Gene DNA kit (Qiagen). Trio‐based WES was performed with NextSeq500 sequencing instruments (Illumina) according to the standard procedures as previously described.^24^, ^25^, ^26^ We adopted a case‐by‐case analytical approach to identify candidate causative variants in each trio. Firstly, we prioritized the rare variants with a minor allele frequency <0.005 in the 1000 Genomes Projects, Exome Aggregation Consortium, and Genome Aggregation Database (gnomAD, gnomad.broadinstitute.org). We retained potentially pathogenic variants, including frameshift, nonsense, canonical splice site, initiation codon, and missense variants predicted as being damaging in silico tools (VarCards, http://varcards.biols.ac.cn/). Finally, we analyzed the potential disease‐causing variants in each case under the following five models: (1) epilepsy‐associated gene model^27^; (2) de novo variant dominant model; (3) autosomal recessive inheritance model, including homozygous and compound heterozygous variants; (4) x‐linked model; (5) co‐segregation analysis model. To identify novel epilepsy‐associated gene, we put the known epilepsy‐associated genes aside, and selected the genes with de novo variants, biallelic variants, hemizygous variants, and variants with segregations for further studies to define the gene‐disease association, as we recently reported.^28^ *CELSR3* emerged as one of the candidate genes with recurrent de novo variants and variants with segregations in this cohort of patients. Conservation of mutated positions was evaluated using sequence alignment of different species. Sanger sequencing was used to validate the positive findings and the variant origination. All *CELSR3* variants identified in this study were annotated to reference transcript NM_001407.3.

### 2.3 Protein modeling

To evaluate the detrimental effect of candidate variants, we performed protein models using Swiss‐Model web server (https://swissmodel.expasy.org/) and Iterative Threading ASSEmbly Refinement (I‐TASSER, https://zhanglab.ccmb.med.umich.edu/I‐TASSER/). The three‐dimensional structures of protein model were displayed by PyMOL (Version 1.6; Schrödinger, LLC).

### 2.4 Statistical analysis

A gene‐based burden analysis^29^, ^30^, ^31^ for *CELSR3* was performed in GraphPad Prism 8. Statistical significance was assessed using two‐sided Fisher's exact test in alleles of cases with FS/EFS+ versus controls (gnomAD) with exact 95% confidence intervals (CIs). *p* < 0.05 was considered significant.

## 3 RESULTS

### 3.1 **Identification and analysis of** *CELSR3* **variants**

**FIGURE 1:**
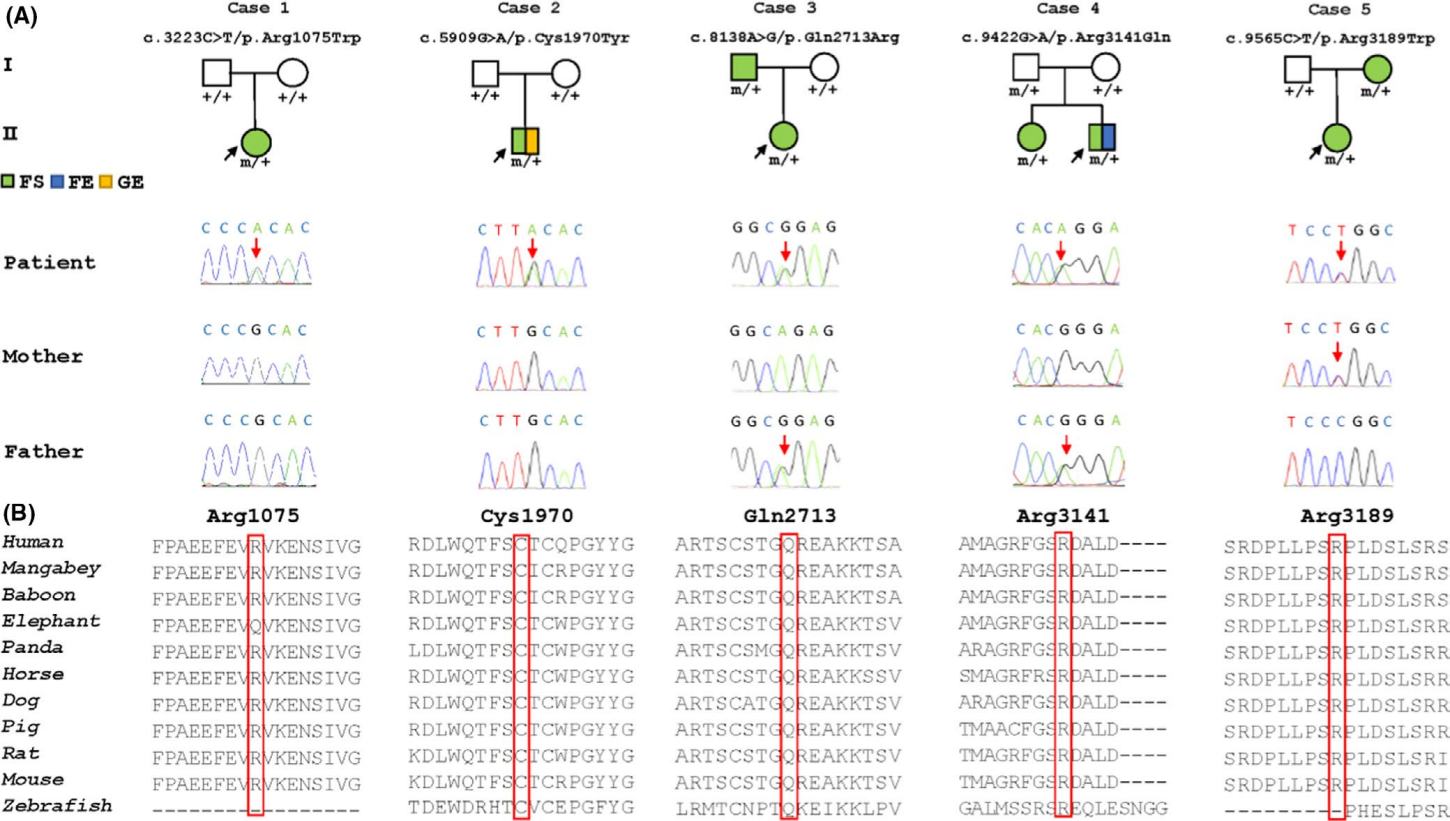
Genetic data of the cases with *CELSR3* variants. (A) Pedigrees of the five cases with *CELSR3* variants, and DNA sequence chromatogram of the *CELSR3* variants. The patients with seizures are represented by solid square/circle, including febrile seizures (FS) colored green, focal epilepsy (FE) colored blue, and generalized epilepsy (GE) colored yellow. Individuals with heterozygous variant are marked by m/+, and those negative for variant are marked by +/+. (B) The amino acid sequence alignment of variants shows that residues Cys1970, Gln2713 and Arg3141 are highly conserved across vertebrates. Residue Arg3189 is conserved across mammalia, whereas residue Arg1075 is less conserved among elephants than other mammalian species

Five heterozygous missense variants in *CELSR3* were detected in five unrelated cases with FS/EFS+, including c.3223C>T/p.Arg1075Trp, c.5909G>A/p.Cys1970Tyr, c.8138A>G/p.Gln2713Arg, c.9422G>A/p.Arg3141Gln, and c.9565C>T/p.Arg3189Trp (Figure 1A, Table 1). Two variants (c.3223C>T/p.Arg1075Trp and c.5909G>A/p.Cys1970Tyr) were de novo. The other three were identified in families with more than one individual affected. The amino acid sequence alignment showed that residues Cys1970, Gln2713, Arg3141, and Arg3189 were highly conserved throughout class mammalia (Figure 1B). Residue Arg1075 was less conserved in elephant, but it was conserved by prediction of GERP++ program (http://mendel.stanford.edu/sidowlab/downloads/gerp/index.html). All the variants were predicted to be damaging in more than one of the commonly used *silico* prediction tools (VarCards, http://varcards.biols.ac.cn/).

**TABLE 1:** Clinical features of cases presented febrile seizures/epilepsy with antecedent febrile seizures with *CELSR3* variants

Case	Variants	Gender	Age (years)	Seizure onset	Diagnosis	Seizure course	Seizure‐free duration	Neurodevelopmental disorders	Treatment	EEG	Brain MRI
Case 1	c.3223C>T (p. Arg1075Trp)	Female	4	2 years	FS	FS, only once during whole course	2 years	None	None	Normal	Normal
Case 2	c.5909G>A (p. Cys1970Tyr)	Male	3	1 year	FS, GE	FS and aFS (GTCS), 1/month^a^ for 1 year; 2 SE	1 year	None	LEV	Normal	Normal
Case 3‐1	c.8138A>G (p. Gln2713Arg)	Female	5	1 year 6 months	FS	FS, 2–4/year^a^ for 2 year	1 year 6 months	None	None	Normal	Normal
Case 3‐2		Male	34	1 year	FS	FS, 3/year^a^ for 1 year	32 years	None	None	n.a.	n.a.
Case 4‐1	c.9422G>A (p. Arg3141Gln)	Male	6	2 years	FS, FE	FS and aFS (sGTCS), 1–5/year^a^ for 3 year; 3 SE	1 year	None	LEV, VPA	Bilaterally posterior spike‐slow waves	Normal
Case 4‐2		Female	8	3 years	FS	FS, 2/year^a^ for 1 year	4 years	None	None	n.a.	n.a.
Case 5‐1	c.9565C>T (p. Arg3189Trp)	Female	8	3 years	FS	FS, 1–2/year^a^ for 3 year	2 years	None	None	Normal	Normal
Case 5‐2		Female	36	2 years	FS	FS, 2/year^a^ for 3 year	31 years	None	None	n.a.	n.a.

Abbreviations: aFS, afebrile seizures; EEG, electroencephalogram; FE, focal epilepsy; FS, febrile seizures; GE, generalized epilepsy; GTCS, generalized tonic‐clonic seizures; LEV, levetiracetam; n.a., not available; MRI, magnetic resonance imaging; SE, status epilepticus; sGTCS, secondarily generalized tonic‐clonic seizures; VPA, valproic acid.

^a^ Frequency of seizures.

Variant p.Cys1970Tyr was not present in gnomAD database. The frequencies of variants p.Arg1075Trp, p.Gln2713Arg, p.Arg3141Gln, and p.Arg3189Trp were far below 0.001 in gnomAD (Table 2). A gene‐based burden analysis for *CELSR3* variants was performed between the cases with FS/EFS+ and the controls in gnomAD database (Table 3). Five variant alleles were detected in the present cohort (462 cases, 924 alleles, 5/924); in contrast, these variant alleles presented at a frequency of 42/277466 and 23/19828 in the controls of all population (gnomAD) and East Asian population (gnomAD), respectively. Statistically significant differences were detected between the case group and control groups (5/924 vs. 42/277466, *p* < 0.000001, odds ratio (OR) [95% CI] = 35.75 [14.11–90.56]; 5/924 vs. 23/19828, *p* = 0.007368, OR [95%CI] = 4.665 [1.922–11.91]).

**TABLE 2:** Genetic features of cases presented febrile seizures/epilepsy with antecedent febrile seizures with *CELSR3* variants

Case	Coordinate (hg19)	cDNA change (NM_001407.3)	Protein change	Inheritance	MAF‐All	MAF‐East Asian	SIFT	PP2_HDIV	PP2_HVAR	Mutation Taster	CADD	GERP++	phyloP	phastCons	SiPhy
1	chr3:48696845	c.3223C>T	p. Arg1075Trp	De novo	2.84 × 10^−5^	0	T (0.081)	P (0.993)	P (0.683)	PM (1.0)	D (23.4)	C (2.53)	NC (0.419)	NC (0.000)	NC (6.669)
2	chr3:48689324	c.5909G>A	p. Cys1970Tyr	De novo	‐	‐	D (0.000)	P (1.0)	P (0.995)	D (1.0)	D (28.2)	C (5.72)	C (7.523)	C (1.000)	C (16.849)
3	chr3:48681676	c.8138A>G	p. Gln2713Arg	Paternal^a^	1.22 × 10^−5^	2.00 × 10^−4^	T (0.182)	B (0.173)	B (0.241)	D (0.999)	D (23.4)	C (2.65)	C (4.433)	C (1.000)	NC (7.660)
4	chr3:48677596	c.9422G>A	p. Arg3141Gln	Paternal	7.42 × 10^−5^	8.00 × 10^−4^	D (0.007)	P (0.991)	P (0.62)	D (0.686)	D (34)	C (4.92)	C (3.311)	NC (0.438)	NC (11.595)
5	chr3:48677453	c.9565C>T	p. Arg3189Trp	Maternal^a^	8.24 × 10^−6^	5.83 × 10^−5^	D (0.001)	P (0.996)	P (0.736)	PM (0.994)	D (26.7)	NC (−0.54)	NC (1.752)	NC (0.994)	C (13.583)

Note

B: benign; C: conserved; D: damaging; P: possibly damaging; T: tolerable.

Abbreviations: CADD, combined annotation dependent depletion; MAF, minor allele frequency from gnomAD_exome; NC, nonconserved; PM, polymorphism; PP2, polyphen2.

^a^ Affected parent.

**TABLE 3:** A gene‐based burden analysis for *CELSR3* variants identified in febrile seizures/epilepsy with antecedent febrile seizures

	Allele count/number in this study	Allele count/number in the controls of gnomAD‐all population	Allele count/number in the controls of gnomAD‐East Asian population
Identified *CELSR3* variants			
c.3223C>T/p.Arg1075Trp	1/924 (0.0011)	10/282,850 (0.000035)	0/19,954 (0)
c.5909G>A/p.Cys1970Tyr	1/924 (0.0011)	‐/‐	‐/‐
c.8138A>G/p.Gln2713Arg	1/924 (0.0011)	4/281,468 (0.000014)	4/19,938 (0.0002)
c.9422G>A/p.Arg3141Gln	1/924 (0.0011)	23/278,086 (0.000083)	18/19,848 (0.00091)
c.9565C>T/p.Arg3189Trp	1/924 (0.0011)	5/277,466 (0.000018)	1/19,828 (0.00005)
Total	5/924 (0.0054)	42/277,466 (0.00015)	23/19,828 (0.0012)
*p*‐value		<0.000001	0.007368
OR (95% CI)		35.75 (14.11–90.56)	4.665 (1.922–11.91)

Note

*p*‐value was determined by two‐sided Fisher's exact test, alleles in patients versus controls; The value [95% CI] was calculated by method of Woolf logit.

Abbreviations: CI, confidence interval; OR, odds ratio.

None of the eight affected individuals were detected any other pathogenic or likely pathogenic variants in the 977 genes known to be associated with epilepsy.^27^

### 3.2 Structural alteration of CELSR3 protein

**FIGURE 2:**
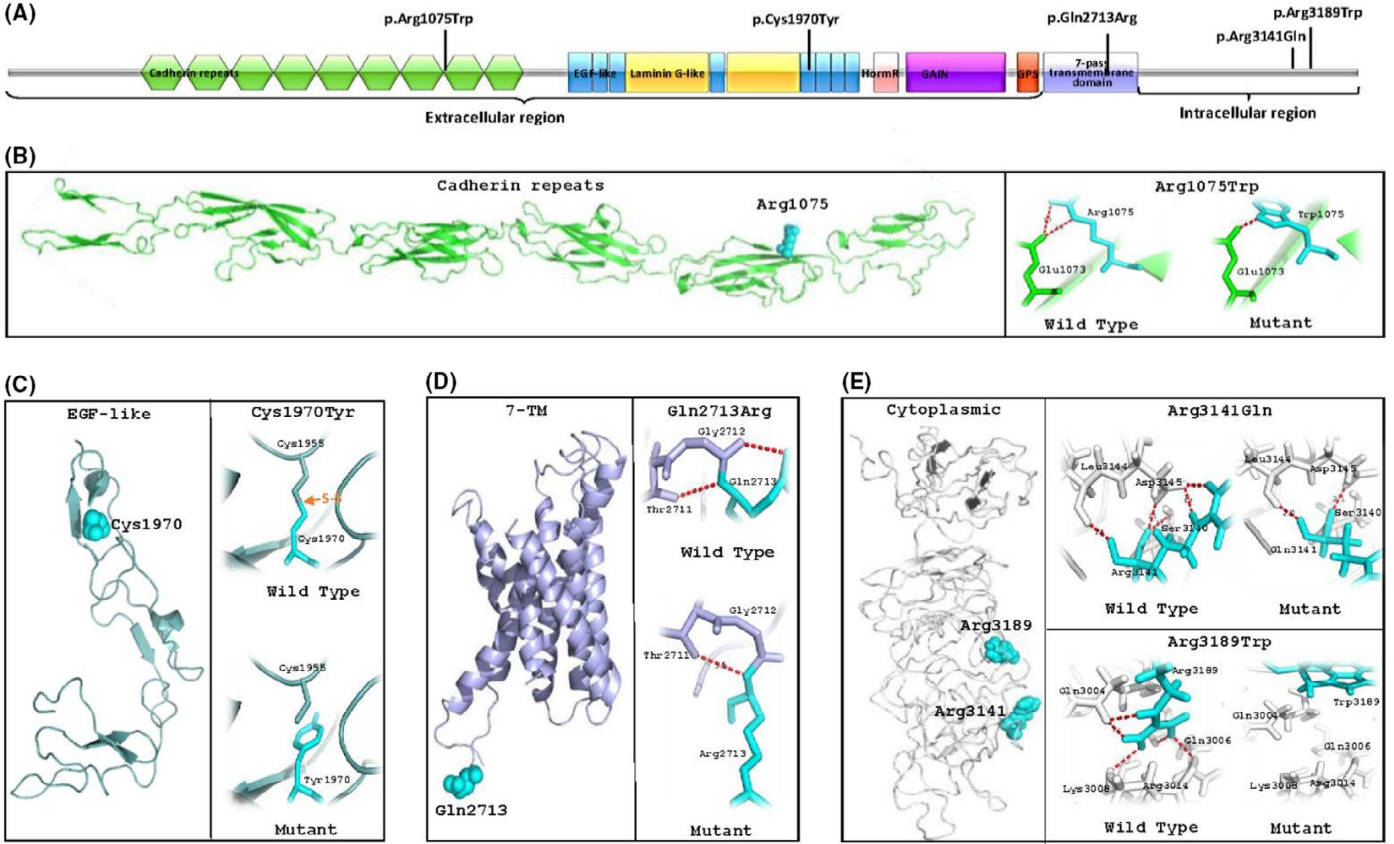
Schematic presentation of CELSR3 structure. (A) Linear representation of the subunit polypeptide chain and the location of CELSR3 variants. CELSR3 is a modular structure, including an extracellular region, a transmembrane domain, and an intracellular region. The extracellular region is composed of nine cadherin repeats (colored green), eight epidermal growth factor‐like repeats (EGF‐like) (colored blue), two laminin A G‐type repeats (colored yellow), and one GPCR proteolytic site (GPS) (colored red). The transmembrane domain (colored purple) is formed by seven‐transmembrane helices. The intracellular region contains a cytoplasmic tail. Variant Arg1075Trp (located on nine cadherin repeats) and variant Cys1970Tyr (located on EGF‐like domain) are both in extracellular region. Variant Gln2713Arg is located on transmembrane domain. Variant Arg3141Gln and variant Arg3189Trp are both in intracellular region. (B–E) Schematic illustration in the three‐dimensional structure of CELSR3, including part of cadherin repeats (B), EGF‐like domain (C), transmembrane domain (D), and topological domain of intracellular region (E). (B) Residue Arg1075 (showed by cyan spheres) is on the outer side of cadherin repeats and links residue Glu1073 with two hydrogen bonds. In the mutant, only one hydrogen bond between Trp1075 and Glu1073 is kept at a distance of 1.9 Å. (C) Residue Cys1970 (showed by cyan spheres) is on the outer side of EGF‐like domain, linking residue Cys1955 with a disulfide bond (showed by orange arrow). In the mutant, the disulfide‐link is destroyed when residue Cys1970 is replaced by Tyr1970. (D) Residue Gln2713 (showed by cyan spheres) is located on the outer side of transmembrane domain, which forms two hydrogen bonds with Thr2711 and Gly2712. In the mutant, the residue Arg2713 forms one hydrogen bond with Thr2711 and destroys the hydrogen bond with Gly2712. (E) Residue Arg3141 and residue Arg3189 are both located on the outer side of topological domain in intracellular region. Residue Arg3141 forms five hydrogen bonds with residue Ser3140, Leu3144, and Asp3145. In mutant Arg3141Gln, two hydrogen bonds with Leu3144 and Asp3145 are kept, while the other three hydrogen bonds are destroyed. Residue Arg3189 interacts with residue Gln3004, Gln3006, Lys3008, and Arg3014 by forming five hydrogen bonds. When arginine at residue 3189 is replaced by tryptophan, all linked hydrogen bonds are destroyed

CELSR3 contains a large extracellular region for calcium binding, a seven‐transmembrane domain in charge of stabilizing the extracellular structure and transducing signal, and an intracellular region serving as a docking point for downstream effectors in cytoplasm (Figure 2A).^6^ Variants Arg1075Trp and Cys1970Tyr were both in the extracellular region of CELSR3, locating at the 9th cadherin tandem repeat domain (cadherin 9) and the EGF‐like, respectively. Variant Gln2713Arg located at the seven‐transmembrane domain. Variants Arg3141Gln and Arg3189Trp were both at the topological domain of intracellular region (Figure 2A).

Three‐dimensional structural model of CELSR3 showed that the affected residues were all located on the outer side of the domains (Figure 2B–E). Originally, residue Arg1075 formed two hydrogen bonds with Glu1073 at distances of 3.0 Å and 3.3 Å, respectively. When arginine was replaced by tryptophan at residue 1075, one of the hydrogen bonds with residue Glu1073 was destroyed and the other hydrogen bond was shortened to a distance of 1.9 Å (Figure 2B). Residue Cys1970 linked Cys1955 with a disulfide bond, which was destroyed as cysteine at residue 1970 being replaced by tyrosine (Figure 2C). Residue Gln2713 formed two hydrogen bonds with Gly2712 and Thr2711. When glutamine was replaced by arginine at residue 2713, the hydrogen bond with Gly2712 was destroyed (Figure 2D). Residue Arg3141 formed five hydrogen bonds with Ser3140, Leu3144, and Asp3145. When arginine was replaced by glutamine, three hydrogen bonds were destroyed while the two hydrogen bonds with Leu3144 and Asp3145 were kept. Residue Arg3189 interacted with Gln3004, Gln3006, Lys3008, and Arg3014 by forming five hydrogen bonds. When arginine at residue 3189 was replaced by tryptophane, all hydrogen bonds above were destroyed (Figure 2E).

### 3.3 Clinical features of the cases with *CELSR3* variants

In this study, the *CELSR3* variants were identified in five cases (families) with eight individuals (five females, three males) affected (Figure 1A, Table 1). All affected individuals were diagnosed with FS/EFS+, including six individuals with FS, one patient with generalized tonic‐clonic seizures with antecedent FS, and one patient with secondarily generalized tonic‐clonic seizures with antecedent FS. Two probands (case 2 and case 4‐1) experienced status epilepticus. The age at seizure onset ranged from 1 year old to 3 years old (mean 1 year 11 months [standard deviation] 9 months). The frequencies of seizures in all cases were low, mostly being at 1–5 times per year. Four cases were normal on EEG recordings, and one case (case 4‐1) had diffused spike‐slow waves with posteriorly dominant. The brain MRI were all normal. With a minimum one‐year follow‐up, all cases had favorable outcomes. Six patients were seizure‐free without medication, while two patients (case 2 and case 4‐1) were seizure‐free after ASMs therapy. No patients had developmental delay, intellectual disability, and other significant neurological issues.

## 4 DISCUSSION

CELSR3 is a cell adhesion protein and largely expressed in the brain, functioning as a signaling receptor. CELSR3 converts cell–cell communication cues into intracellular signals and regulates nervous activity.^6^ In our study, heterozygous *CELSR3* variants were identified in five unrelated cases (families) with eight affected individuals from a cohort of 462 patients with FS/EFS+. The identified *CELSR3* variants included two de novo variants and three missense variants affected more than one individual in families. All variants were predicted to be potentially damaging by silico tools. The gene‐based burden analysis showed that the frequency of *CELSR3* variants identified in this study was significantly higher than that in controls. Protein modeling implied conformational space changes of the *CELSR3* variants, which potentially caused functional impairments of the protein.^32^ These findings suggest that *CELSR3* variants are potentially associated with FS/EFS+.

In mammals, CELSR3 was expressed in brain from prenatal period. Homozygous *CELSR3* KO mice exhibited severe development abnormality of nervous system in embryonic period and died with neuropathological anomalies few hours after birth,^10^ suggesting that *CELSR3* played a key role in early neurodevelopment of mammals. In the present study, all patients carried heterozygous *CELSR3* variants and presented mild clinical symptoms with favorable outcomes. Protein modeling showed that the variants resulted in disappearance of multiple hydrogen bonds and one disulfide bond, but the affected residues were all located on the outer side of domains. Generally, the residues on the outer side of protein interconnected with fewer residues than central residues in structure; mutant residues at the outer side of domain had less effects on the protein structure and function than those at the inner side.^33^ It was, therefore, considered that the mild phenotypes were potentially due to the less damage effects caused by the heterozygous variants that located on the outer side of domains. It was possible that heterozygous variants might lead to milder phenotypes, whereas biallelic variants were lethal, leading to absence of clinical phenotypes.

Febrile seizures is a common clinical phenotype in humans, particularly in East Asian populations. In this study, the aggregate frequency of the *CELSR3* variants was 0.0054 in the present cohort with FS/EFS+, which was significantly higher than in controls of all population and East Asian population (gnomAD), suggesting an association between *CELSR3* variants and FS/EFS+. It was noted that the frequency of *CELSR3* variants in East Asian populations was higher than that in all populations (Table 3). The incidence of FS was higher in East Asian populations,^34^ which could potentially be explained by the higher frequency of FS‐related variants like *CELSR3* in East Asian populations.

In previous studies, three homozygous *CELSR3* variants were reported, including two homozygous *CELSR3* variants (c.6407G>A/p.Gly2136Asp^21^ and c.7890G>A/p.Met2630Ile^20^) identified in patients with early infantile epileptic encephalopathy. The variant Met2630Ile presented at a frequency of 0.4% in general population, and 11 homozygous Met2630Ile presented in the controls of population (gnomAD), suggesting that the variant Met2630Ile was not pathogenic likely. The variant Gly2136Asp presented at a frequency of 0.002% in general population, and no homozygous Gly2136Asp presented in the controls of population. Both the patients with Met2630Ile and Gly2136Asp had homozygous *CACNA2D2* variants, which was a disease‐causing gene of early‐onset epileptic encephalopathy/global developmental delay and potentially explained the epileptic encephalopathy. Therefore, the association between homozygous *CELSR3* variants and epileptic encephalopathy was uncertain. A homozygous *CELSR3* variant was identified in a case with intellectual disability.^16^ On the other hand, eighteen heterozygous *CELSR3* variants have been detected in patients with neuropsychiatric disorders, including thirteen cases with neural tube defects,^12^, ^14^, ^17^ two cases with developmental delay/intellectual disability,^15^, ^18^ two cases with Tourette syndrome,^13^ and one case with autism spectrum disorder^19^ (Table S1). Five of the heterozygous variants were of de novo, and thirteen of the variants were of unknown origin. Taken together that homozygous *CELSR3* KO mice exhibited severe development abnormalities of the nervous system and neonatal death, it was possible that patients with homozygous *CELSR3* variants of severe damage would be dead in early life, whereas patients with homozygous *CELSR3* variants of less severe damage or with heterozygous *CELSR3* variants of severe damage would present neurodevelopmental disorders, and patients with heterozygous *CELSR3* variants of mild damage would present FS and/or epilepsy. The phenotypical spectrum of *CELSR3* variants potentially ranges from mild FS and/or epilepsy to neurodevelopmental disorders or even early death, depending on the dose of gene damage,^24^ which warrants further verification.

There are several limitations in this study. First, functional study on *CELSR3* variants was not performed. *CELSR3* encodes a large protein consisted of 3312 amino acids, which brought challenges on functional experiments of in vitro. Second, *CELSR3* variants are potentially associated with other neurodevelopmental disorders, such as neural tube defects, developmental delay/intellectual disability, and autism spectrum disorders. The present study focused on epilepsy, which is potentially one of the phenotypes within the spectrum of *CELSR3* variants. Future studies are required to determine the whole spectrum of *CELSR3* variants.

In conclusion, we identified five heterozygous *CELSR3* variants in five unrelated cases (families) with eight individuals affected by FS/EFS+. All patients presented favorable outcomes without neurodevelopmental disorders. The gene‐based burden of variants showed a significant association between *CELSR3* variants and FS/EFS+. Protein modeling suggested that the variants led to structure alterations of CELSR3 but were located on the outer side of the domains, potentially explaining the mild phenotype. These findings suggest that *CELSR3* variants are potentially associated with FS/EFS+.

## CONFLICT OF INTEREST

The authors have stated that they had no interests that might be perceived as posing a conflict or bias.

## Supporting information

Supplementary Material

## Data Availability

Data available on request from the authors.
