# Supplementary material for: CELSR3 variants are associated with febrile seizures and epilepsy with antecedent febrile seizures
Source: CNS Neurosci Ther. 2021 Dec 23;28(3):382–9. doi: 10.1111/cns.13781 (PMC8841303; doi:10.1111/cns.13781)
Supplement: Supplementary file 1 — Supplementary Material [file CNS-28-382-s001.docx]

| **TABLE S1 *CELSR3* variants detected in neurological disorders** | | | | |
| --- | --- | --- | --- | --- |
| **Type of genetic variant** | **Variant** | **MAF-All** | **Phenotype** | **Reference** |
| **Homozygous** |  |  |  |  |
|  | c.2951C>T/p.Ser984Leu | 5.00×10^-4^ | ID | Harripaul et al., 2018 |
|  | c.6407G>A /p.Gly2136Asp | 2.14×10^-5^ | EE | Pippucci et al., 2013 |
|  | c.7890G>A/p.Met2630Ile^†^ | 4.00×10^-3^ | EE | Edvardson et al., 2013 |
| **Heterozygous** |  |  |  |  |
|  | **missense variant** |  |  |  |
|  | c.581G>T/p.Gly194Val | - | NTD | Qiao et al., 2016 |
|  | c.598G>A/p.Gly200Ser | 4.07×10^-6^ | NTD | Qiao et al., 2016 |
|  | c.1060G>A/p.Ala354Thr | - | NTD | Qiao et al., 2016 |
|  | c.1336G>A/p.Val446Met | - | NTD | Qiao et al., 2016 |
|  | c.1865C>T/p.Ala622Val (*de novo*) | - | ASD | Jiao et al., 2019 |
|  | c.2348G>A/p.Arg783His (*de novo*) | 1.22×10^-5^ | Tourette syndrome | Willsey et al., 2017 |
|  | c.3304A>T/p.Ile1102Leu | 8.43×10^-6^ | NTD | Chen et al., 2018 |
|  | c.3581G>A/p.Arg1194His | 4.06×10^-5^ | NTD | Chen et al., 2018 |
|  | c.4357C>T/p.Arg1453Cys | 3.26×10^-5^ | NTD | Chen et al., 2018 |
|  | c.5260G>A/p.Gly1754Ser | 2.03×10^-5^ | NTD | Chen et al., 2018 |
|  | c.5261G>A/p.Gly1754Asp | 0 | NTD | Qiao et al., 2016 |
|  | c.7751T>G/p.Val2584Gly | 2.47×10^-5^ | NTD | Chen et al., 2018 |
|  | c.7890G>A/p.Met2630Ile | 4.00×10^-3^ | NTD | Chen et al., 2018 |
|  | c.8335C>G/p.Arg2779Gly | - | NTD | Ishida et al., 2018 |
|  | c.8587G>A/p.Ala2863Thr | 8.16×10^-6^ | NTD | Qiao et al., 2016 |
|  | c.8786G>T/p.Arg2929Leu (*de novo*) | - | ID, DD | Vissers et al., 2017 |
|  | **non-missense variant** |  |  |  |
|  | c.5634+1G>T/- (*de novo*) | NA | DD | Zhu et al., 2015 |
|  | c.5706dupC/p.(Gly1903Argfs*107) (*de novo*) | NA | Tourette syndrome | Willsey et al., 2017 |

^†^: Eleven homozygous variants presented in all populations (gnomAD).

ASD: autism spectrum disorder; DD: developmental delay; FE, focal epilepsy; FS: febrile seizures; GE, generalized epilepsy; ID: intellectual disability; MAF, minor allele frequency from gnomAD_exome; NA: not available; NTD: neural tube defects.
